# Noninvasive Diagnostics of Renal Amyloidosis: Current State and Perspectives

**DOI:** 10.3390/ijms232012662

**Published:** 2022-10-21

**Authors:** Sergei A. Fedotov, Maria S. Khrabrova, Anastasia O. Anpilova, Vladimir A. Dobronravov, Aleksandr A. Rubel

**Affiliations:** 1 Laboratory of Amyloid Biology, St. Petersburg State University, St. Petersburg 199034, Russia; 2 Pavlov Institute of Physiology, Russian Academy of Sciences, St. Petersburg 199034, Russia; 3 Research Institute of Nephrology, Pavlov University, St. Petersburg 197101, Russia; 4 Department of Genetics and Biotechnology, St. Petersburg State University, St. Petersburg 199034, Russia

**Keywords:** protein aggregation, amyloids, early diagnostics, proteinuria, serum, kidney biopsy, chronic kidney disease, seeding

## Abstract

Amyloidoses is a group of diseases characterized by the accumulation of abnormal proteins (called amyloids) in different organs and tissues. For systemic amyloidoses, the disease is related to increased levels and/or abnormal synthesis of certain proteins in the organism due to pathological processes, e.g., monoclonal gammopathy and chronic inflammation in rheumatic arthritis. Treatment of amyloidoses is focused on reducing amyloidogenic protein production and inhibition of its aggregation. Therapeutic approaches critically depend on the type of amyloidosis, which underlines the importance of early differential diagnostics. In fact, the most accurate diagnostics of amyloidosis and its type requires analysis of a biopsy specimen from the disease-affected organ. However, absence of specific symptoms of amyloidosis and the invasive nature of biomaterial sampling causes the late diagnostics of these diseases, which leads to a delayed treatment, and significantly reduces its efficacy and patient survival. The establishment of noninvasive diagnostic methods and discovery of specific amyloidosis markers are essential for disease detection and identification of its type at earlier stages, which enables timely and targeted treatment. This review focuses on current approaches to the diagnostics of amyloidoses, primarily with renal involvement, and research perspectives in order to design new specific tests for early diagnosis.

## 1. Introduction

Amyloidoses is a heterogeneous group of diseases, which results from proteins misfolding with the formation of amyloid aggregates accumulated in tissues with subsequent progressive organ dysfunction and failure [1,2]. Amyloids are fibrils of orderly complexed proteins that are connected by hydrogen bonds to form an intermolecular cross-β-structure [3,4]. Amyloid fibrils are typically composed of two or more protofilaments or, in some cases, of a single protofilament. Protofilaments are connected to each other in a parallel fashion via their side chains [5]. Each protofilament has a cross-β structure, where β-strands are stacked perpendicular to the fibril axis [6]. Amyloids usually bind specific dyes, such as Thioflavin T (ThT) and Congo red [7,8,9], and exhibit resistance against the actions of proteases and various detergents [10,11]. The amyloidogenic properties may be a consequence of variations in the amino acid sequence and are manifested in different pathologies associated with increased levels of amyloidogenic protein [1,12,13,14]. Current classification of amyloidoses is based on the type of protein that predominantly forms fibrils in the deposits [5]. More than 30 amyloidogenic proteins and peptides have been found that accumulate in organs and tissues and cause approximately 70 different forms of amyloidoses in humans, with the majority being extremely rare [5,15].

The triggers of amyloidogenesis are not yet fully discovered. In the case of localized amyloidoses, individual organs may be affected by local production of amyloid protein in skin, soft tissues, urinary bladder, digestive tract, respiratory tract, and others [16]. 

**Figure 1:**
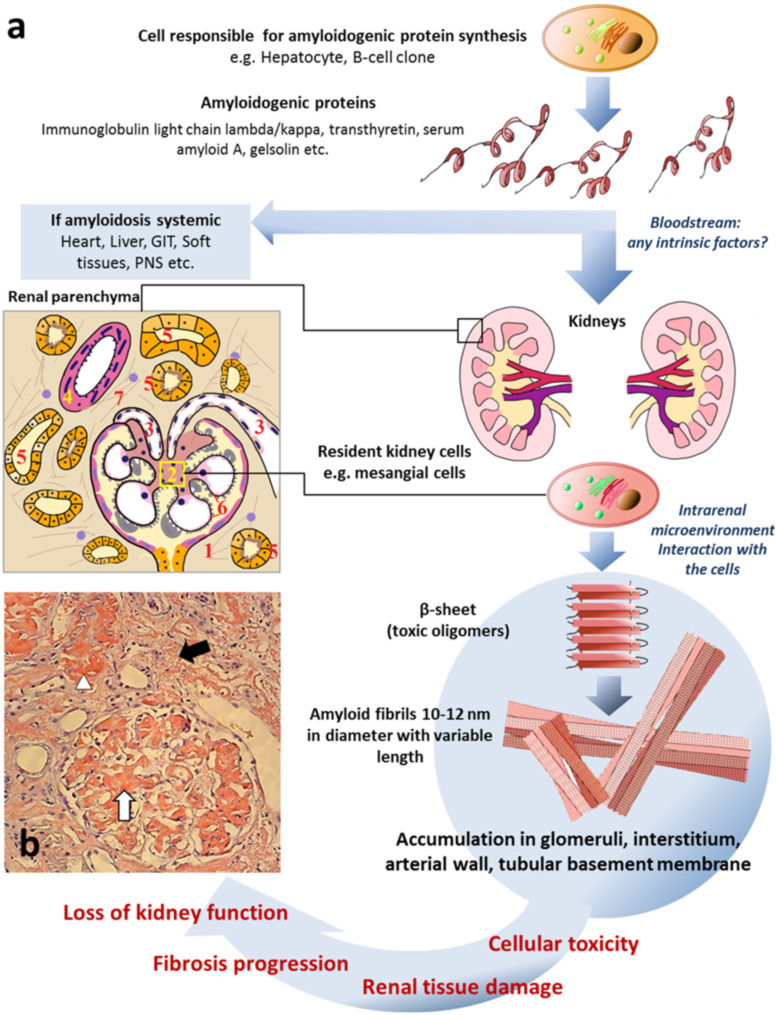
Common mechanisms of renal amyloidosis. (**a**) Schematic representation of process of renal amyloidosis formation. GIT—gastrointestinal tract; PNS—peripheral nervous system; scheme of renal parenchyma: 1—glomerulus; 2—mesangium; 3—arterioles; 4—arterial wall; 5—tubules; 6—glomerular basement membrane; 7—interstitium. (**b**) The microphotograph demonstrating amyloid deposition in mesangium, capillary basement membranes and arterioles of glomerulus (white arrow), interstitium (black arrow) and arterial wall (arrowhead) presented as homogenous Congo-positive masses. Congo red stain, original magnification ×200 (the microphotograph was obtained by V.G. Sipovsky.

The circulation of amyloidogenic proteins in systemic disease leads to amyloid deposition in multiple tissues throughout the body [17] (Figure 1). Systemic amyloidoses have a worse prognosis than the localized form of disease [18]. The most common types of systemic amyloidosis such as immunoglobulin (Ig) light chain (AL) amyloidosis, hereditary and wild-type transthyretin (ATTR) amyloidosis, serum amyloid A (AA) amyloidosis and leukocyte chemotactic factor 2 (ALECT2) amyloidosis cause progressive organ dysfunction and end-stage kidney disease [19,20,21]. The incidence of systemic amyloidosis exceeds 0.8/100,000 of the population [22].

In the context of pathological processes, several diseases of the central nervous system (CNS), such as Alzheimer’s diseases and Parkinson’s diseases, form a specific distinct category in the amyloidosis entity. These disorders could result from the toxic effect of soluble oligomeric amyloid aggregates, rather than structural tissue alterations due to amyloid deposits, as it shown in cell culture and animal models [23,24]. Cellular toxicity of non-fibrillar (oligomeric) protein forms are also suggested for AL and ATTR amyloidoses [12,19,25].

The diagnosis of the amyloidosis type is critical for assigning therapies, reducing the specific protein level in the body, and inhibiting the pathological processes associated with this protein [26,27]. The effectiveness of the treatment largely depends on the stage at which the disease is diagnosed [28,29,30]. According to the current clinical criteria, the diagnosis of amyloidosis can be verified only by a morphological analysis of impaired organ biopsy samples, e.g., kidney biopsy in the case of renal amyloidosis [31]. The gold standard for diagnosis of amyloidosis is the positive staining of tissue specimens to amyloid-specific Congo red dye with apple green birefringence under polarized light microscopy [32,33].

Currently, there are different approaches based on immunodetection and mass spectrometric technologies that enable the type of amyloidosis to be determined by analysis of Congo red positive biopsy specimen (for review, see [34]). Current clinical practice based on clinical and histological presentation leaves many patients with systemic amyloidosis undiagnosed, especially those with earlier stages of the disease. Therefore, new approaches for amyloidosis confirmation and determination of its type are warranted. The only noninvasive method with 100% specificity and positive predictive value is based on infusion of ^99m^Tc-labeled bone scintigraphy tracers and their accumulation in the myocardial amyloid deposits. This imaging technique, in fact, has been designed exclusively for the detection of cardiac ATTR amyloidosis in the absence of plasma cell dyscrasia [35]. Radioactively-labeled serum amyloid P component (SAP) can be applied for amyloid assessment, but only at a rather late stage, where massive tissue deposits are already apparent [36]. This method can hardly be used for differentiation of amyloidosis type (AA or AL) due to considerable overlap between patterns of organ involvement [36]. Hence, improvement in noninvasive diagnostics of amyloidosis and its types represents a significant clinical problem. The identification of new diagnostic markers for different amyloidosis types has clinical relevance, while being important for understanding the pathogenesis of the disease as well.

There have been previously published reviews considering methods of early noninvasive diagnosis of CNS amyloidosis and their significance for the effective monitoring and management of the disease [37,38,39]. This review focuses on the current advances and new approaches in noninvasive diagnostic modalities for systemic amyloidosis, primarily with renal involvement. 

## 2. Different Types of Renal Amyloidosis

In systemic amyloidoses, kidney involvement is known to be very frequent and usually manifests with nephrotic syndrome and chronic kidney disease [19]. Nowadays, about 15 amyloid proteins are known to impair the kidneys (Table 1) with the pooled prevalence of AL, ALECT2, or AA types exceeding 88% [21]. The less common variants of renal amyloidosis are characterized by an accumulation of deposits of fibrinogen α chain, Ig heavy chains, apolipoproteins, and other precursor proteins (Table 1), as well as co-aggregation of Ig heavy and light chains [21,31]. Additionally, cases of two types of amyloidosis co-existing have also been described, either co-localizing in the same organ or affecting different organs [31,40]. Each type of amyloidosis has specific features depending on the affected renal structures (e.g., glomeruli, tubulointerstitium, and arteries) and the distribution of the renal deposits (diffuse or focal) [41]. Conversely, the wide range of morphological alterations and corresponding clinical manifestations may be observed at the same amyloidosis type [42,43]. In addition to kidney abnormalities, patients often have extrarenal disease manifestations (Table 1) [44,45]. Amyloid deposition in myocardium is associated with inferior patient survival [46], meanwhile kidney amyloid deposition results in end-stage kidney disease that requires renal replacement therapy [47,48].

AL-amyloidosis is the most common type of systemic amyloidosis in developed countries [22] and mainly relates to organ deposition of aberrant Ig kappa (Igκ) or Ig lambda (Igλ) light chains produced by the malignant clone of B-cell lineage (plasmacytic, lymphoplasmacytic, or lymphocytic). Due to numerous genetic changes, this clone co-produces light chain molecules with a destabilized structure of Ig variable domain [53]. The aggregation-prone state of the Ig variable domain of monoclonal protein is considered to be responsible for the progression of AL-amyloidosis [1]. 

AA-amyloidosis is another common type of renal amyloidosis [12], characterized by the tissue accumulation of serum amyloid A protein (SAA) aggregates. Increased SAA production is a response to chronic inflammation (rheumatoid arthritis, Crohn’s disease, chronic osteomyelitis, bronchiectasis, etc.). Other common types are hereditary and senile forms of ATTR amyloidoses, ATTRv and ATTRwt, respectively [54,55], caused by the accumulation of transthyretin protein (TTR). TTR accumulation primarily affects the heart, and only in rare cases, is ATTR associated with renal abnormalities [31]. ALECT2 and AFib amyloidoses are associated with deposition of leukocyte chemotactic factor 2 and fibrinogen α chain precursor proteins, respectively. New data indicate that ALECT2 amyloidosis may have a substantially higher prevalence than previously suspected. According to [21], ALECT2 accounts for 19.1% of the renal amyloidosis cases and yields only to AL amyloidosis. ALECT2 amyloidosis is most common in the Hispanic population [56] and primarily targets the kidneys among the elderly. AFib amyloidosis leads especially to end-stage kidney disease in the elderly, and the deposit formation is associated with a mutation in the *FGA* gene [57]. Amyloidoses associated with apolipoproteins and other proteins such as gelsolin and lysozyme do not involve kidneys. 

## 3. Tissue- and Cell-Based Diagnostics of Amyloidosis

Early diagnostics of systemic amyloidosis is crucial as the disease has unfavorable prognosis if left untreated [22]. The median survival has been found to be about 6–11 years in AA amyloidosis [26,58] and about 1–3 years in AL amyloidosis [59,60] with the tendency toward declining mortality in the era of new treatment modalities [61]. Current available therapeutic options depend on the pathogenesis of particular amyloidosis type. The limitation of amyloid protein production by targeting specific pathological processes is the basic treatment approach. Such an approach, in particular, is used to reduce SAA levels by suppressing inflammation in rheumatoid arthritis, tuberculosis, periodontal disease, etc. [62]. A decrease in production of aberrant Ig light chains in AL-amyloidosis can be achieved by clone-directed chemotherapy, with or without autologous stem cell transplantation [63]. The treatment of hereditary ATTR amyloidosis is accomplished with liver transplantation, as the liver is a primary source of systemic TTR pool [64], and by prescribing functional tetramer TTR stabilizers [65,66]. The use of antisense oligonucleotides or silencing TTR mRNA has also found to be effective in phase 3 clinical trials [67,68]. Further efforts are in progress for AL amyloidosis in order to create stabilizers of the native dimeric structure of full-length Ig light chains or siRNA to reduce pathological λ-light-chain production [69,70]. Moreover, a number of amyloid-clearing immunotherapeutic agents are being clinically tested. Antibody targeting of amyloid proteins have not been proven as yet to be effective in systemic amyloidosis while studies on doxycycline treatment provided promising results (for review, see [27]).

The therapeutic efficacy is critically dependent on the stage of disease. The high mortality in AL amyloidosis [71] to a great extent refers to the difficulties in early diagnostics [72]. Disease recognition is complicated because clinical presentation of amyloidosis is largely non-specific and similar to that in other disease [73]. As a result, patients with amyloidosis often remain misdiagnosed and subjected to prolonged evaluation and unwarranted treatment. The assumption of amyloidosis is based on systemic organ damage, including nephrotic syndrome, unexplained cardiac dysfunction, autonomic or sensory neuropathies, periorbital purpura, weight loss, and other signs unrelated to the specific pathological processes [74]. 

In order to assess the degree of organs involvement and to clarify the type of amyloidosis, a number of noninvasive imaging tools could be used, such as echocardiography, magnetic resonance imaging, and imaging of radioactive tags injected into the circulation [75,76]. SAP protein with a radioactive tag, for instance, is trapped in amyloid deposits, thus making it possible to detect the localization of deposits by the radioactive signal. This method provides a high level of sensitivity and specificity (>90%) for the diagnosing of amyloidosis and determining the localization of the deposits in the body [36], however, without specifying the protein composition of the deposits. Important sensitive, although not specific, biomarkers of AL amyloidosis, are N-terminal pro-B-type natriuretic peptide (NT-proBNP) for the heart, proteinuria for the kidney, and alkaline phosphatase (ALP) for the liver. AL amyloidosis may occur in the presence of multiple myeloma (MM) most often being associated with monoclonal Igλ or λ free light chains (FLC) in the serum [63].

Suspected diagnosis of systemic amyloidosis with renal involvement should be proven by a morphological analysis of a kidney specimen. Alternatively, in a case of contraindications for kidney biopsy, one may consider an analysis of extrarenal tissue such as bone marrow, liver, and abdominal fat (for review, see [34]). However, the diagnostic value of non-renal samples is lower in comparison to renal ones [47,77].

Kidney biopsy specimens are studied using light microscopy, immunoassays, and electron microscopy. Special Congo red staining shows detected amyloid material as a red color under light microscopy and as apple green birefringence under polarized light as a result of dye binding with amyloids [32,33]. Immunofluorescence microscopy of frozen sections and immunogold labeling or immunohistochemistry (IHC) of formalin-fixed paraffin-embedded (FFPE) sections using antibodies against Igκ and Igλ, SAA, TTR and other amyloidogenic proteins make it possible to determine the type of amyloidosis [34]. The diagnostic effectiveness of IHC using antibodies against amyloid precursor proteins depends significantly on the quality of reagents, pathologist experience [78], and often requires antigen retrieval for antibodies in pretreatment with protease digestion, microwave heating, pressure cooking, and others [79]. In the case of AL amyloidosis, there is a possibility of false-negative results due to the presence of variable domains in Ig light chains [31], which can probably affect the folding of the proteins in aggregates and the epitope availability. Amyloids can include both full-length proteins and single fragments of Ig light chains, which also influences the efficiency of immunodetection [80,81] and the ability to specify the type of light chains, Igκ or Igλ [82]. In contrast, false-positive results are problematic for the diagnosis of AA amyloidosis [83]. At the same time, automatic assay systems with 100% sensitivity and specificity in diagnosing amyloidosis using an optimized antibody panel have been recently developed [84]. Immunoelectron microscopy (IEM) employing gold-labeled secondary antibodies provides more specificity in comparison to other antibody-based techniques due to the ability to detect nanostructures (amyloid fibrils and non-amyloid complexes) to which antibodies bind [85,86] and thereby exclude background staining.

The most effective approach for diagnosing amyloidosis and its type is laser microdissection followed by tandem mass spectrometry (LMD–MS) of biopsy tissue specimens [87]. Proteins isolated from Congo red-stained FFPE sections are tryptically digested into peptides that are sequenced by mass spectrometry (MS). This method also provides the identification of common amyloidosis marker proteins such as SAP and apolipoprotein E (amyloid signatures), for which its presence in samples serves as an internal control of procedure and confirms amyloid deposits [49,88]. LMD–MS tissue analysis provides the opportunity to correctly identify the amyloidosis type with sensitivity and specificity up to 100% [89,90,91]. It should be noted that the LMD–MS-approach has demonstrated greater efficiency compared to immunoassays for the identification of amyloidosis type on specimens from the same batch. According to [91], LMD–MS of FFPE affected organ biopsy samples enabled identification of amyloidosis type in 92% of cases, whereas the use of antibodies provided detection in only 45% of cases on specimens from the identical sampling. Gilbertson et al. demonstrated 100% concordance between positive IHC and LMD–MS in 142 sequential biopsy samples from 38 different tissue types; however, the diagnostic accuracy was 76% and 94%, respectively [90]. Gonzalez Suarez reported that immunofluorescence staining for Igκ or Igλ has inferior sensitivity and specificity compared with LMD–MS in the typing of 170 cases of renal immunoglobulin-derived amyloidosis [92]. Identification of 12.3% of cases failed in the immunofluorescence assay. LMD–MS is also an essential method for identifying extremely rare forms of amyloidosis [21]. Along with LMD–MS, there are other MS-based proteomics methods demonstrating their advantage over antibody-based techniques. The study by [93] reported that 2D-PAGE-based comparative proteomics allowed the identification of amyloidosis type in two cases in which the IEM assay was unsuccessful. However, immunoassays are still the most common diagnostic technique in clinical laboratories because the technique is more reproducible and lower in cost than LMD–MS.

Mutation studies for various hereditary amyloidosis are performed to verify the amyloidosis type identified by biopsy analysis and also to refine the disease predicting [94]. Specifically, ATTRv amyloidosis are related to the presence of point mutations in the *TTR* gene including Val30Met, Val122Ile, Thr60Ala, and AFib amyloidosis is caused by point mutations (most common Glu526Val) or frameshift mutations in the *FGA* gene [95,96]. When AA amyloidosis due to systemic autoinflammatory disease is suspected, the diagnostics can be complemented with genetic testing on mutations in *MEFV*, *NLRP3*, *MVK*, and other genes [97]. A current list of mutations could be viewed at http://amyloidosismutations.com/ (accessed on 19 September 2022) [98]. Genetic analysis is essential, as hereditary amyloidosis may mimic AL in immunoassays [99]. Notably, genetic analysis requires caution because the penetrance of mutations can be highly variable [57,100]. A more reliable method to verify the hereditary amyloidosis type (sensitivity 92% and specificity 100%) is mass spectrometric detection of variant proteins isolated from biopsy specimens [101].

Thus, the analysis of biopsy-derived material is an effective method of diagnosing the amyloidosis type. However, kidney biopsy is generally performed by indication in the case of pronounced organ dysfunctions and higher risks of treatment failure. Another major disadvantage of this approach is the invasive nature of the procedure and risk of complications that limit its use in certain cases.

## 4. Noninvasive Evaluation of Amyloidosis and Its Types

A major area of noninvasive diagnostics is the development of scanning research methods (radiography, computed tomography, ultrasound, magnetic resonance imaging, bone scintigraphy, and others). Currently, the application of those methods is limited to the late stages of amyloidosis in the presence of the significant deposition of amyloid aggregates in the organs [102,103,104]. Therefore, imaging is mainly applied to verify the amyloidosis diagnosis and clarify the type of amyloidosis. For example, ^123^I-labeled SAP scintigraphy provides detection of either AA- or AL- amyloidosis types with 90% sensitivity. However, this approach has limited value because of overlapping patterns of organ involvement for both types of amyloidosis [36]. Injection of ^99m^Tc-labeled bone scintigraphy tracers into circulation followed by the visualization of their accumulation in myocardial amyloid deposits provides specificity and a positive predictive value of 100% for the diagnosis of ATTR cardiac amyloidosis, but only in the absence of plasma cell dyscrasia [35]. At the same time, plasma cell dyscrasia is a common competing disease, especially in the elderly, and was detected in 39% of ATTRwt cases and in 49% of patients with ATTRv amyloidoses [105]. Positron emission tomography and computed tomography with thioflavin-analogue tracers [106,107] enable the detection of Ig light chain deposits in organs not previously identified by clinical manifestations or biopsy. While being effective for the detection of asymptomatic amyloid in some extra-renal organs, PET identified renal involvement in fewer subjects than the international consensus diagnostic approach was able to [108]. Research applying diffusion-weighted magnetic resonance imaging, which is sensitive to local water motion in the tissue, becomes more promising for the detection of amyloid nephropathy [102]. However, the sensitivity and specificity of that method in the detection of amyloidosis are still low (79% and 60%, respectively). Collectively, imaging methods continue to be only a supporting tool in the diagnosis of renal amyloidosis.

Another area of noninvasive diagnosis is the analysis of body fluids (saliva, blood, urine, mucous membrane epithelial scrapes), which are collected without damaging the internal organs affected by amyloidosis. This approach provides opportunities for detecting factors and disease markers before the occurrence of severe systemic abnormalities that are recalcitrant to therapy. Fluid analysis, firstly, simplifies the condition monitoring of patients with already diagnosed amyloidosis and, secondly, enables the screening of the early stages of disease until progressive systemic abnormalities become apparent.

Since the 1970s, there have been several attempts to detect amyloid aggregates in the urine. Several studies have demonstrated the presence of different types of fibrillar structures in the urine in amyloidosis [109,110]. This approach, however, has been called into question by the fact that fibrils were not found in all specimens from patients with amyloidosis, and were also observed in control specimen groups [111,112,113].

Nowadays, there are a variety of methods for detecting the precursor protein of amyloidosis in serum and urine. Palladini et al. demonstrated that immunofixation electrophoresis of serum and urine (IFE) performed with anti-IgG, -IgA, -IgM, -Igκ, and -Igλ antibodies on gels could identify the amyloidogenic Ig light chains in all 115 patients with a monoclonal gammopathy [114]. The serum FLC immunoassay used for calculating the Igκ/Igλ ratio had less sensitivity (76%). However, a combination of these two methods had a 100% sensitivity [114]. Igκ/Igλ ratio outside of the physiological range (0.26–1.65) with high urine albumin levels implicates renal AL amyloidosis [115], and increased serum NT-proBNP values indicate cardiac AL amyloidosis in the absence of echographic features of heart involvement [116]. Despite the methods reaching 100% sensitivity in the detection of amyloidosis, they are nonspecific and require further verification of the diagnosis. Ig light chain is found in 100% of cases with other types of monoclonal gammopathies, including light chain monoclonal gammopathy of undetermined significance (MGUS) [117], which is significantly more prevalent than AL amyloidosis [118]. As noted above, monoclonal light chains could be found in the serum in patients with ATTRwt, AA, and other amyloidosis types [105,119]. On the contrary, increased levels of precursor protein SAA (>10 mg/mL) are associated with the progression of established AA amyloidosis in most cases [120]. However, SAA concentrations have no value for the prediction of this amyloid type incidence [121] and, hence, cannot substitute standard histological diagnostics.

The possibility of detection of precursor proteins in biological fluids by MS is currently being studied (for review, see [122]), despite the nonspecificity of the approaches to the identification of precursor proteins in fluids. These methods frequently involve the use of antibodies to enrich samples with putative precursor proteins because of the high content of non-target proteins in plasma and urine specimens (in the case of proteinuria) [123,124,125]. Using the MS provides an opportunity to detect the monoclonal protein in the serum and urine at concentrations below the threshold values for the IFE and FLC assay [124,126]. The MS of serum samples for ATTR amyloidosis enables the diagnosis of inherited forms of that disease [127]. The detection of peptides in serum and plasma samples with specific post-translational modifications at Cys-10 by liquid chromatography–MS is considered to be a promising approach for the diagnosis of ATTRv amyloidosis [123].

New perspectives in the analysis of precursor proteins of AL amyloidosis include the assessment of the Ig light chain functional properties detected in body fluids. The glycosylation of precursor proteins, their toxicity, and the genes encoding the monoclonal protein provide a high-level confidence in predicting of amyloidosis in a group of patients with MGUS, smoldering myeloma, or MM [13,128,129,130,131]. Proteomic analysis of urine exosome can be used for the differential diagnosis of renal disorders in monoclonal gammopathies [132]. In particular, in renal AL amyloidosis out of remission, immunoreactive proteins are found in urine exosomes corresponding to λ light chains, in contrast to non-amyloid renal injury in MM [132]. The use of the nematode *Caenorhabditis elegans* as an object for testing the toxicity of a monoclonal protein (“biosensor”) has demonstrated the possibility of diagnosing the cardiac AL amyloidosis in MM patients by the level of pathogenic effects on the pharynx of nematode [13]. Amyloidogenic Ig light chains isolated from the urine and serum of patients with cardiac AL amyloidosis caused cell death in the pharynx and reduced pumping rate. In contrast, non-cardiotoxic Ig light chains from patients with renal amyloidosis and MM had no effect on organ function [17]. Kumar et al. [131] found the presence of N-glycosylation of Ig light chains in 33 of 189 samples obtained by immunoprecipitation from the serum of patients with AL amyloidosis. Glycosylation of Igκ was detected for 32.8% of AL patients, and glycosylation of Igλ–for 10.2% of cases. The glycosylation rate in patients who had a detected monoclonal protein but without of AL amyloidosis was only 4.1% (5 of 122 cases) [131]. As a result, the risk group screening for Ig light chains glycosylation has a positive predictive value of 86.8%, which is extremely high for a noninvasive approach. Subsequent study has indicated that glycosylation in the MGUS group makes it possible to assess the risks of AL amyloidosis. Rates of progression at 20 years were 21% and 3% for AL patients with and without glycosylated light chains, respectively [129]. Kumar et al. also reported that glycosylation more frequently affects sites of polypeptides encoded by genes of the KV1 and LV3 families [131]. It has been previously demonstrated that proteomics-determined Ig germline gene usage provides a risk assessment of organ damage in AL amyloidosis [130]. Light chain variable region (IGVL) genes *LV6-57*, *LV3-01*, and *LV3-21* are associated with renal injury, *LV1-44* is more frequently identified in patients with cardiac AL amyloidosis, *LV2-14* and *KV1-33* usage are more probable if peripheral nerve and liver, respectively, are involved [130]. At the same time, in another recent study of Ig germline gene usage by cDNA analyses IGLV1-44 was the most dominant IGLV-subfamily for patients with dominant kidney involvement and IGLV3-21 with dominant heart involvement [128]. These and other inconsistencies indicate that the clinical significance of the identification of IGVL genes encoding the monoclonal protein in patients with AL amyloidosis requires further evaluation.

**Figure 2:**
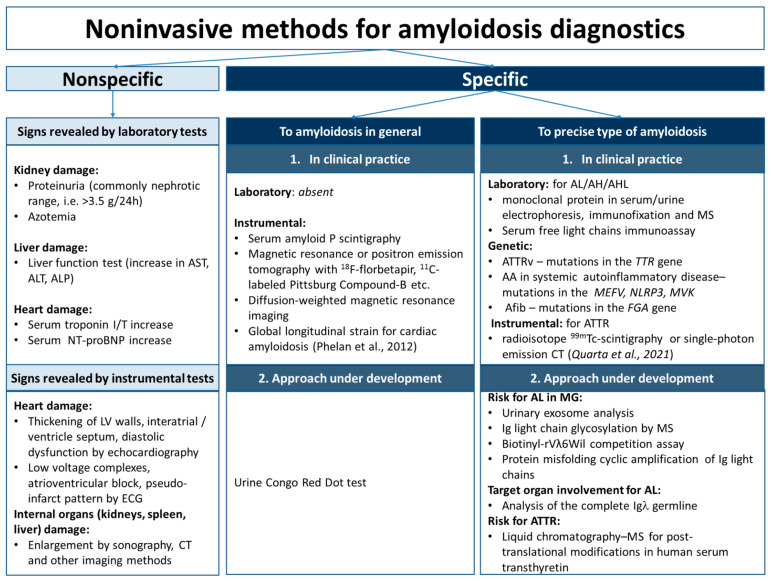
Noninvasive methods for amyloidosis diagnostics. AA—serum amyloid A amyloidosis; AFib—fibrinogen α chain amyloidosis; AH—immunoglobulin heavy chains amyloidosis; AHL—immunoglobulin heavy and light chains amyloidosis; AL—immunoglobulin light chains amyloidosis; ALP—alkaline phosphatase; ALT—alanine aminotransferase; AST—aspartate aminotransferase; ATTRv—hereditary type of transthyretin amyloidosis; CT—computed tomography; ECG—electrocardiography; I/T—troponin I/troponin T; LV, left ventricle; MG—monoclonal gammopathy; MS—mass spectrometry; NT-proBNP—N-terminal pro-B-type natriuretic peptide [133,134].

Thus, the available range of noninvasive methods in the amyloidosis evaluation seems to be useful in clarifying the organ involvement, assessment prognosis, and disease monitoring (Figure 2). However, these methods do not allow us to make an ultimate diagnosis of amyloidosis per se and to ascertain its type.

## 5. Promising Trends in Noninvasive Diagnostics

**Figure 3:**
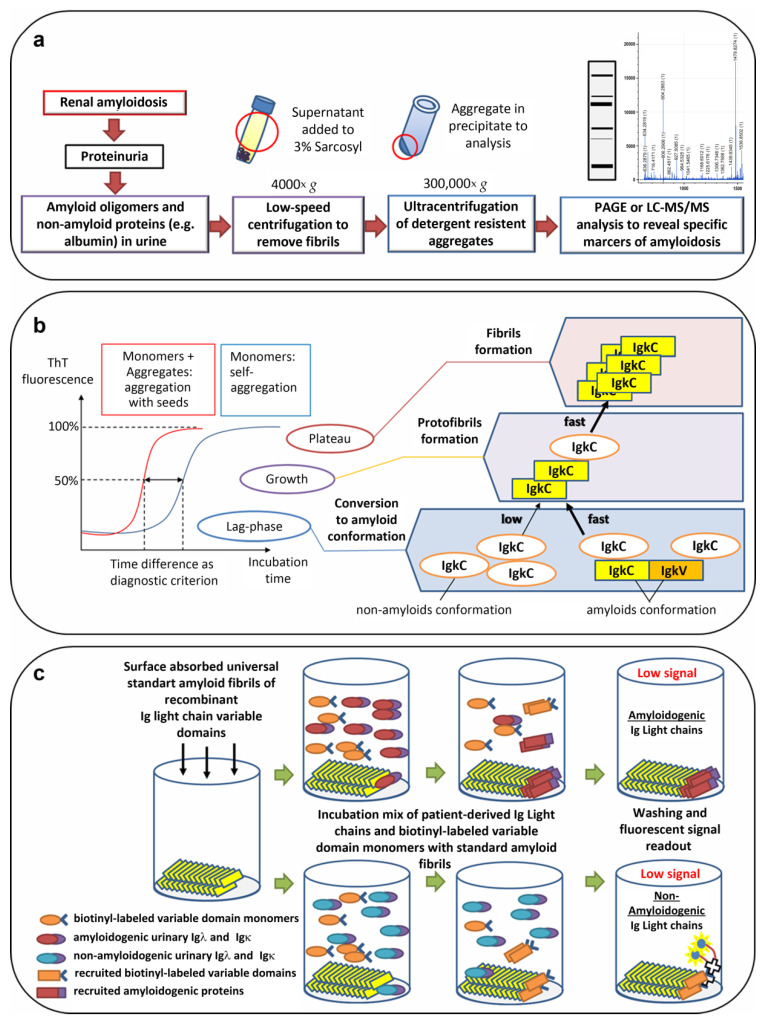
Perspective approaches for noninvasive diagnostics of amyloidoses. (**a**) Methods based on the amyloid resistance to detergents. (**b**) Seeding of amyloidogenic protein monomers by preexisting aggregates. (**c**) Recruitment of amyloidogenic monomer proteins by synthetic amyloid fibrils. PAGE—polyacrylamide gel electrophoresis; LC-MS/MS—liquid chromatography with tandem mass spectrometry; ThT—thioflavin; IgκC—Igκ constant domain; IgκV—Igκ variable domain.

In the absence of noninvasive methods of early diagnosis for the majority of amyloidosis, it is necessary to carry out research with new approaches. One of the trends is to use specific properties of amyloid aggregates and precursor proteins. These properties include the seeding of amyloidogenic protein monomers by preexisting aggregates [135], amyloid resistance to detergents and proteases [10,136], and the recruitment of amyloidogenic monomer protein by synthetic amyloid fibrils [137] (see Figure 3). 

Amyloid aggregates, characterized by the presence of an array of β-strands, are known to induce and promote the formation of new aggregates by monomers of these proteins (nucleation and secondary seeding processes, for review, see [138]). The addition of aggregates to the monomer solution and periodic fragmentation of nascent fibrils leads to exponential growth of aggregated protein forms, making the detection of amyloids in biological fluids possible even at extremely low concentrations [139,140]. Both of these and related amyloid amplification methods are abbreviated as PMCA, from Protein Misfolding Cyclic Amplification, or QuIC, from Quaking-Induced Conversion. New-forming fibrils, typically, can be detected by Thioflavin T (ThT), the fluorescent dye. Previously, use of urine samples as seed in PMCA demonstrated high sensitivity for diagnosing diseases associated with amyloid deposition of prion proteins in the brain [141]. It is known that the Ig light chains isolated from the urine of patients with amyloidosis are capable of aggregation in vitro, as are prion proteins [142]. Alternatively, [143] reported that the ex vivo fibrils extracted from the heart of patient with cardiac AL amyloidosis accelerated fibril formation of homologous and non-homologous monomers of Igλ variable domain. The acceleration of time-resolved ThT fibrillation kinetics with ex vivo fibrils as seeds has also been demonstrated for TTR and SAA proteins [136,144]. Until now, no one has reported the possibility of detecting minor amounts of these protein aggregates in the urine or blood by PMCA or QuIC. In the future, since PMCA-based detection frequently demonstrates high sensitivity, that approach could provide detection of fibrils and specific oligomeric forms of pathological Ig light chains variants scarcely represented in the biological fluids of patients with amyloidosis.

One of the challenges in applying the protein amplification approach to AL amyloidosis is the unique nature of each Ig light chain due to the complementarity-determining regions of the variant domain. This problem could be solved by selection of the monomeric protein required to be induced by the aggregates from the biological fluids. The amino acid sequence of the selected protein will differ from the sequence of Ig light chains and their fragments in aggregates obtained from different patients. Therefore, the amplification reaction must proceed through cross-seeding between heterologous proteins [145]. Blancas-Mejía et al. show that the time of Igκ variable domain aggregation upon the addition of aggregates obtained in vitro from domains with a different sequence (sequence identity with the *IGKV 1–33* germline gene 91–96%) varies significantly in various combinations [146]. Cross-seeding most often caused the acceleration of monomer aggregation, but the lag phase reduction could vary considerably for different monomeric proteins. The lag-phase reduction in cross-seeding was occasionally shorter compared to homologous seeding. Moreover, for one variant of the aggregates, an inhibition of the amplification rate during the cross-seeding of a monomeric protein variant was observed [146]. The authors generally conclude that the efficiency of seeding is determined mainly by the amyloidogenic properties of the monomers in solution rather than by the properties of the seed. Notably, the addition of aggregates formed by Igκ variable domains had no effect on the aggregation kinetics of monomeric variable domains from the λ1b subgroup (sequence identity with the germline gene *IGKV 1-33* 48%) [146].

Another interesting finding of [146] was the fact that aggregates of the germline gene *IGKV 1-33* product can accelerate fibril formation in a solution of full-length Igκ monomers. That result raises the issue of considering the use of Igκ constant domain monomers for the detection of amyloidogenic monoclonal proteins in biological fluids. Since the constant domain is unchanged in all Igκ variants except for rare mutations, its use can provide a stable seeding with lower effects on aggregation kinetics varying between samples. It was demonstrated that monomers of the constant domain of Igκ light chains exhibit in vitro self-aggregation with the forming of amyloid fibrils [147]. In addition, amyloid aggregates formed by Igκ constant domain were described [148]. The successful use of protein fragments for amplification of aggregates in biological sample has been shown previously for the detection of human Creutzfeldt–Jakob disease using cerebrospinal fluid [149]. Amyloidogenic Ig light chains as monomers, oligomers, and as a part of aggregates can also possibly promote faster conversion of constant domain monomers to amyloid conformation both through direct interaction with constant domains in aggregates and through cross-seeding by amyloidogenic sites in the variable part of light chains [145]. 

A different approach for the optimization of monoclonal protein amyloidogenicity assessments is the development of universal standard aggregates of Ig light chains. For the testing amyloidogenicity of Ig light chains, [137] used synthetic amyloid fibrils composed of a λ6 variable domain isolated from an AL patient (rVλ6Wil). Four samples of AL-associated radiolabeled urinary Igλ and Igκ bound rVλ6Wil fibrils (recruitment) more efficiently than four samples of MM-associated proteins. Furthermore, in MM patients with abnormally high Ig light chain recruitment, AL amyloidosis was subsequently diagnosed, thus suggesting the prognostic potential of the proposed methodological approach. Notably, the AL-associated protein recruitment to Aβ(1–40) fibrils was more pronounced in comparison to MM proteins. The method was further improved by using biotinyl-λ6 variable domain monomers in solution together with patient-derived urinary proteins [150]. Competitive binding to rVλ6Wil fibrils between recombinant λ6 variable domain and urinary proteins permitted separation of MM and AL patient groups with 100% specificity and sensitivity by concentration-dependent inhibition of biotinyl-λ6 variable domain recruitment [150].

The assessment of amyloidogenicity of protein components and detection of amyloid aggregates in biological fluids are complicated by the presence of a large number of other proteins (not participating in amyloidogenesis). Specifically, albumin, the most abundant protein in serum and in urine samples from patients with nephrotic syndrome can inhibit amyloid formation [151]. Martin et al. isolated Ig light chains from urine samples [150] following the protocol proposed by [152] in several steps, including dialysis, zone electrophoresis, and gel filtration. In the mass spectrometric studies [124,127,153], the issue of target factor concentration was solved by preliminary immunoprecipitation of proteins suspected for amyloid formation. 

**Figure 4:**
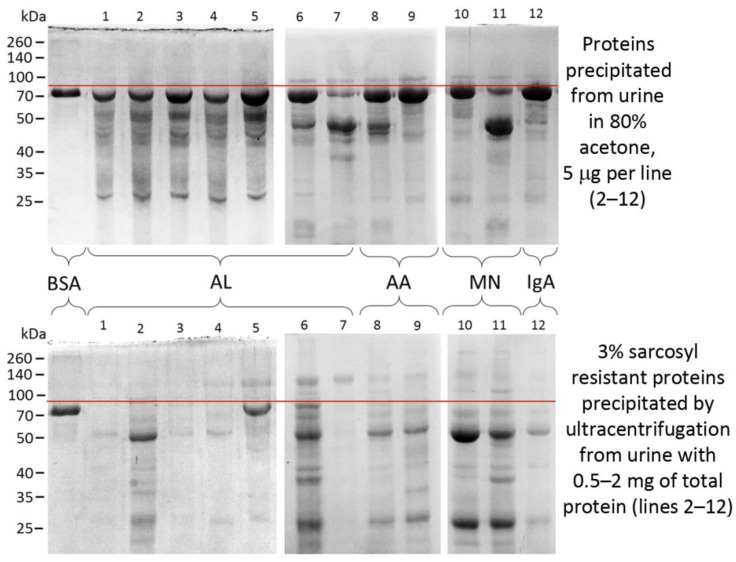
Proteins of urine and detergent-resistant aggregates from patients with proteinuria. Samples are separated in polyacrylamide gel electrophoresis and stained by Coomassie Brilliant Blue. BSA—bovine serum albumin, AL—AL amyloidosis, AA—AA amyloidosis, MN—membranous nephropathy, IgA—immunoglobulin A nephropathy. Spectra Multicolor Broad Range Protein Ladder (no. 26634) (Thermo Fisher Scientific, Waltham, MA, USA) is shown to the left of the gels. Red line indicates the area just above the BSA band (2 µg) that runs at the level of human serum albumin in the samples on the right (lines 1–12). Images was obtained by S.A. Fedotov.

Alternatively, one way to dispose of bulk protein and prepare a sample to assess the amyloidogenicity of the protein components is ultracentrifugation at speeds in the range of thousands *g* and treatment of the deposit with ionic detergents, such as sarcosyl and sodium dodecyl sulfate. It provides detergent-resistant aggregates from the sample and can be used for research and diagnostic purposes [11]. Ultracentrifugation eliminates the background of factors not involved in the aggregate formation, and treatment with detergents enables the avoidance of protein complexes of non-amyloid nature [154]. According to our preliminary data, the quantitative and qualitative composition of proteins in the fraction of aggregates resistant to the treatment with 3% sarcosyl significantly varies in urine samples with different etiologies of proteinuria and significantly differs in protein composition from the untreated samples (Figure 4). 

Generally, the obtained aggregates contain practically no albumin, and preliminary centrifugation at a speed of 4000× *g* enables the removal of large fibrils, the presence of which is not necessarily associated with amyloidogenic factors in the sample and can be found in healthy humans [112]. The analysis of the composition of detergent-resistant aggregates in specimens from patients with various diagnoses may have scientific and clinical relevance, not assessed by anyone to date. This is supported by the data on the detection in isolated urine aggregates the main factors for the progression of preeclampsia, a disease in pregnant women, which, as well as in renal amyloidosis, is associated with proteinuria and amyloid deposits in tissues ([155,156]; see review [157]). Of interest, the Congo red dot (CRD) test and the CRD paper test on urine samples have also demonstrated successful diagnostic applications for preeclampsia [155,158]. The design of those tests is based on the suggestion that Congo red binds amyloidogenic proteins in the urine of preeclampsia patients [155]. Performing CRD tests on the proteinuric specimens of urine in patients with various amyloidoses and non-amyloid diseases could clarify the diagnostic performance of such an approach.

## 6. Conclusions

The current arsenal of diagnostic approaches for renal amyloidosis is based primarily on invasive procedures applying typically at late stages. Since the effectiveness of renal amyloidoses treatment largely depends on the stage at which the disease is diagnosed the design of noninvasive screening techniques for renal amyloidosis might enable earlier detection of the disease, and significantly improve the renal and life prognosis in these patients. In this regard, further study of functional and pathological amyloid properties in humans is essential for the development of new diagnostic and treatment methods for amyloidosis.

## Figures and Tables

**Table 1:** Characteristics of renal amyloidoses [21,31,49,50,51,52].

Amyloidogenic Protein	Sporadic (S) or Hereditary (H)/Local (L) or Systemic (S)	Overall Incidence/Prevalence of Kidney Impairment, %	Extrarenal Manifestation (Other Organ Tropism)
Ig light chains	S/S	59/53–86	Different organs
Serum amyloid A protein	S/S	2.9/7–40	Different organs
Leukocyte cell-derived chemotaxin-2	S/S	3.1/2.5–19.1	Liver, lungs, spleen, adrenal glands, prostate, gastrointestinal tract, gall bladder
Ig heavy chains	S/S	2.3/0.3–4.6	Different organs
Ig light and heavy chains	S/S	not assessed/3.6	Different organs
Fibrinogen α chain	H/S	0.44/1.3–3.5	Liver, spleen, gastrointestinal tract, fat
Transthyretin	S/S; H/S	28/0.9–1.4	Different organs with heart and peripheral nervous system predominance
Apolipoprotein A–IV	H/S	0.35/0.1–1.06	Heart, gastrointestinal tract, skin, lungs
Gelsolin	H/S	0.18/0.8	Cranial nerves, cornea, liver, gastrointestinal tract, skin, heart, breast, pituitary gland
Apolipoprotein C–II	H/L	<0.1/0.6	-
Apolipoprotein A–I	H/S	0.35/0.1–0.5	Liver, heart, skin, larynx, gastrointestinal tract, muscles, peripheral nervous system
Lysozyme	H/S	<0.1/0.16	Lymphatic nodes, liver, gastrointestinal tract, lungs, fat, heart
Apolipoprotein A–II	H/L	<0.1/<0.1 (extremely rare)	-
Apolipoprotein C–III	H/S	<0.1/<0.1 (extremely rare)	Liver, heart, spleen
Calcitonin	S/S	<0.1/<0.1 (extremely rare)	Thyroid gland, fat

## Data Availability

Not applicable.

## Abbreviations

AA	serum amyloid A amyloidosis
AFib	fibrinogen α chain amyloidosis
AH	immunoglobulin heavy chains amyloidosis
AHL	immunoglobulin heavy and light chains amyloidosis
AL	immunoglobulin light chains amyloidosis
ALECT2	leukocyte chemotactic factor 2 amyloidoses
ALP	alkaline phosphatase
ALT	alanine aminotransferase
AST	aspartate aminotransferase
ATTR	transthyretin amyloidosis
ATTRv	hereditary type of transthyretin amyloidosis
ATTRwt	wild-type type of transthyretin amyloidosis
BSA	bovine serum albumin
CNS	central nervous system
CRD	Congo red dot
CT	computed tomography
ECG	electrocardiography
FFPE	formalin-fixed paraffin-embedded
FLC	free light chains
GIT	gastrointestinal tract
I/T	troponin I/troponin T
IEM	immunoelectron microscopy
IFE	immunofixation electrophoresis
Ig	immunoglobulin
IgA	immunoglobulin A nephropathy
IGVL	light chain variable region
Igκ	immunoglobulin kappa light chain
IgκC	immunoglobulin kappa constant domain
IgκV	immunoglobulin kappa variable domain.
Igλ	immunoglobulin lambda light chain
IHC	immunohistochemistry
LMD–MS	laser microdissection followed by tandem mass spectrometry
LV	left ventricle
MG	monoclonal gammopathy
MGUS	gammopathy of undetermined significance
MM	multiple myeloma
MN	membranous nephropathy
MS	mass spectrometry
NT-proBNP	N-terminal pro-B-type natriuretic peptide
PAGE	polyacrylamide gel electrophoresis
PMCA	protein misfolding cyclic amplification
PNS	peripheral nervous system
QuIC	quaking-induced conversion
SAA	serum amyloid A protein
SAP	serum amyloid P component
ThT	thioflavin T
TTR	transthyretin protein
